# Comparative analysis of reconstructed ancestral proteins with their extant counterparts suggests primitive life had an alkaline habitat

**DOI:** 10.1038/s41598-023-50828-4

**Published:** 2024-01-03

**Authors:** Takayuki Fujikawa, Takahiro Sasamoto, Fangzheng Zhao, Akihiko Yamagishi, Satoshi Akanuma

**Affiliations:** 1 Faculty of Human Sciences, Waseda University, 2-579-15 Mikajima, Tokorozawa, Saitama 359-1192 Japan; 2 Department of Applied Life Science, Tokyo University of Pharmacy and Life Sciences, 1432-1 Horinouchi, Hachioji, Tokyo 192-0392 Japan

**Keywords:** Evolution, Experimental evolution, Palaeontology, Protein design, Environmental biotechnology

## Abstract

To understand the origin and early evolution of life it is crucial to establish characteristics of the primordial environment that facilitated the emergence and evolution of life. One important environmental factor is the pH of the primordial environment. Here, we assessed the pH-dependent thermal stabilities of previously reconstructed ancestral nucleoside diphosphate kinases and ribosomal protein uS8s. The selected proteins were likely to be present in ancient organisms such as the last common ancestor of bacteria and that of archaea. We also assessed the thermal stability of homologous proteins from extant acidophilic, neutralophilic, and alkaliphilic microorganisms as a function of pH. Our results indicate that the reconstructed ancestral proteins are more akin to those of extant alkaliphilic bacteria, which display greater stability under alkaline conditions. These findings suggest that the common ancestors of bacterial and archaeal species thrived in an alkaline environment. Moreover, we demonstrate the reconstruction method employed in this study is a valuable technique for generating alkali-tolerant proteins that can be used in a variety of biotechnological and environmental applications.

## Introduction

Ancestral sequence reconstruction (ASR) is a method used to deduce plausible protein sequences that may have been present in extinct organisms^1–6^. Fossil records offer an invaluable source of information about extinct organisms as well as the likely conditions on early Earth. However, no fossil records of ancient organisms that lived more than 3,500 million years ago have been found. The microfossil discovered in the Pilbara region of Western Australia in 1993 has, so far, been recognized as the oldest known fossil on Earth^7^. Initially it was unclear whether these microstructures could have formed via abiotic processes, but more recent evidence supports their biogenicity^8^. However, approaches to study even earlier lifeforms cannot solely rely on the discovery of still older microfossils. It is now possible to approximate the sequences of ancient genes and proteins by comparing those found in modern genomes. This is because the sequence information of the genes and proteins that were present in ancient species has been inherited throughout the evolutionary process. The concept of resurrecting ancient protein sequences was originally proposed by Pauling and Zuckerkandl more than 50 years ago^9^. At that time, the technology required for accurate reconstruction was unavailable, and their assertion was highly speculative. However, this concept has been refined over time, and with the expansion of genome projects it has become possible to infer ancient sequences with a greater degree of confidence^10,11^. Many studies have used ASR to characterize ancient proteins, obtaining information on their physical properties, including thermal stability^12–15^ and substrate specificity^16–21^, as well as to deduce the likely environment for early life^22–25^.

ASR is not only a powerful tool for inferring ancient protein sequences but also for protein engineering, particularly in designing thermally stable proteins. The inferred ancestral amino acid sequence at the root of a phylogenetic tree of 3-isopropyl malate dehydrogenases was introduced into an extant hyperthermophilic enzyme as an amino acid substitution, which resulted in a further thermostabilized enzyme^26^. ASR often yields thermostable proteins, possibly because the ancestral organisms lived in high-temperature environments and their proteins were highly thermostable^27–29^. However, the inferred ancestral sequences tend to converge to the consensus amino acid in many positions, which also contributes to improving the thermal stability of proteins^30–32^. ASR has been criticized because it is biased toward high stability amino acid sequences that are often more thermostable than the consensus sequences^12^. In principle, ASR can predict an ancestral/consensus sequence irrespective of the possible bias present in the input data of extant homologous sequences compared to the consensus method^33^.

Here, we investigated the pH stability of the ancestral nucleoside diphosphate kinase (NDK) and ribosomal protein uS8. NDK plays a crucial role in the regulation of nucleotide metabolism in cells. The enzyme is responsible for catalyzing the transfer of the γ-phosphate group from a nucleoside triphosphate to a nucleoside diphosphate, thereby producing a new nucleoside triphosphate and a new nucleoside diphosphate. This reaction is important for maintaining the overall nucleotide balance, which is necessary for a wide range of cellular processes such as DNA replication, RNA transcription, and energy metabolism. NDK is found in almost all existing organisms and is highly conserved across different species, suggesting its fundamental role in cellular function. The ribosomal protein, uS8, is a small RNA-binding protein and plays a crucial role in the assembly and stability of the ribosome^34^. Molecular phylogenetic analysis together with inference of the ancestral amino acid sequence for NDK and uS8 were previously carried out, which revealed their highly thermostable ancestral forms at near neutral pH^12,35,36^.

In this study, we aimed to investigate the pH profiles of the unfolding temperatures of the ancestral proteins. Intriguingly, ancestral NDKs and uS8s retain their thermostability even under alkaline conditions. Finally, we discuss the potential pH conditions of the ancestral biosphere and the utility of designing alkali-tolerant proteins using ASR for biotechnological or bioremediation applications.

## Results

### Thermal stabilities of extant NDKs as a function of pH

**Figure 1:**
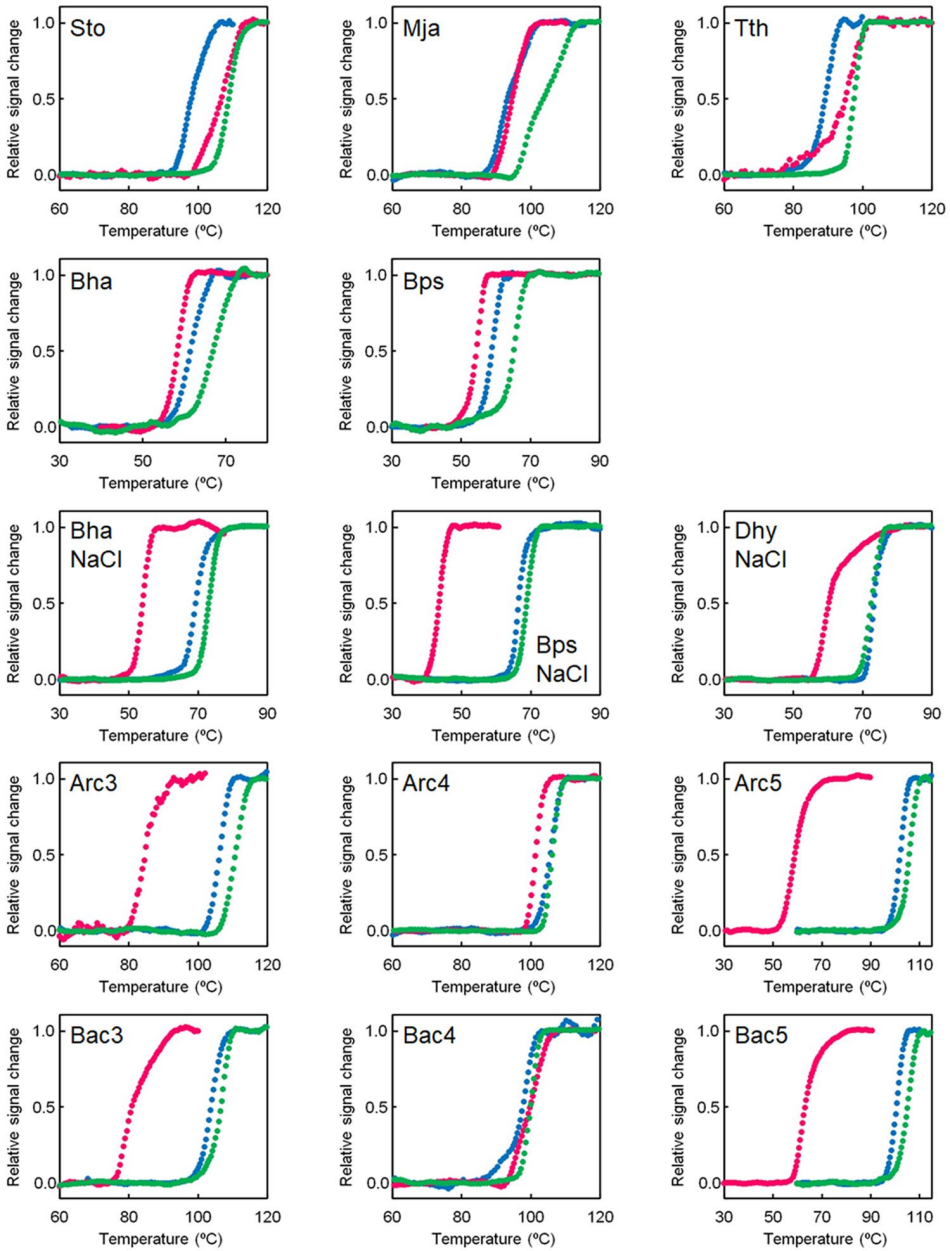
Temperature-induced unfolding curves of extant and ancestral NDKs at pH 5.0 (magenta), pH 7.0 (green) and pH 9.0 (blue). The change in ellipticity at 222 nm was monitored as a function of temperature. Within experimental error, duplicated measurements were identical. The plots were normalized with respect to the baseline of the native and denatured states. Sto, *S. tokodaii* NDK; Mja, *M. jannaschii* NDK; Tth, *T. thermophilus* NDK; Bha, *B. halodurans* NDK; Bps, *B. pseudofirmus* NDK; Dhy, *D. hydrogenovorans* NDK. NaCl indicates that the measurement was carried out in a solution containing 500 mM NaCl.

Initially, the pH-dependent thermal stability of NDKs from extant organisms was analyzed. NDKs from seven organisms were prepared: specifically, *Sulfolobus tokodaii*, *Methanocaldococcus jannaschii*, *Archaeoglobus fulgidus*, *Thermus thermophilus*, *Bacillus halodurans*, *B. pseudofirmus* and *Desulfonatronovibrio hydrogenovorans*. The acidophilic and hyperthermophilic archaeon, *S. tokodaii,* grows optimally at pH 2–3^37^. *M. jannaschii* and *A. fulgidus* are hyperthermophilic archaea and their optimum pH for growth are 6.0 and 6.9, respectively^38,39^. *T. thermophilus* is an extreme thermophilic bacterium and its optimum pH for growth is also nearly 7.0^40^. *B. halodurans*, *B. pseudofirmus* and *D. hydrogenovorans* are alkaliphilic bacteria and grow optimally at pH 10.0–10.5, pH 10.0–11.0 and pH 9.6, respectively^41–43^. The protein from *A. fulgidus* exhibited an unconventional unfolding curve at pH 7.0, indicating a slight increase in the content of helical structure rather than denaturing into a random coil at temperatures between 100 and 106 °C (Fig. S1). Therefore, further thermal unfolding experiments were not performed for *A. fulgidus* NDK. The other extant NDKs were subjected to temperature-induced unfolding experiments at pH values of 5.0, 7.0, and 9.0. To generate the unfolding curves, ellipticity at 222 nm was monitored as a function of temperature. The normalized unfolding curves are shown in Fig. 1. Table 1 lists the unfolding midpoint temperatures (*T*_m_s) at which the relative signal change reaches 0.5 during the unfolding process. These *T*_m_ values were used to compare the thermal stabilities of proteins. The common characteristic shared by acidophilic and neutralophilic NDKs is that their highest *T*_m_s were observed at pH 7.0. In addition, the acidophilic and neutralophilic NDKs have a higher *T*_m_ at pH 5.0 than at pH 9.0.

**Table 1:** *T*_m_ (°C) for ancestral and extant NDKs.

Proteins	*T*_m_ (°C)		
	pH 5.0	pH 7.0	pH 9.0
Ancestral NDKs			
Arc3	85	111	106
Arc4	101	106	106
Arc5	59	106	102
Bac3	81	107	104
Bac4	100	100	98
Bac5	63	105	101
Acidophilic microbial NDK			
*Sto*NDK	107	109	98
Neutralophilic microbial NDKs			
*Mja*NDK	94	104	93
*Tth*NDK	95	98	89
Alkaliphilic microbial NDKs			
*Bha*NDK	58	67	62
*Bha*NDK (500 mM NaCl)	53	73	70
*Bps*NDK	55	65	59
*Bps*NDK (500 mM NaCl)	44	69	67
*Dhy*NDK (500 mM NaCl)	59	73	73

*T*_m_ values were estimated from the data shown in Fig. 1.

Sto *S. tokodaii*, Mja *M. jannaschii*, Tth *T. thermophilus*, Bha *B. halodurans*, Bps *B. pseudofirmus*, Dhy *D. hydrogenovorans.*

The extant alkaliphilic NDKs were subjected to temperature-induced unfolding experiments under conditions of both low (50 mM KCl) and high (500 mM NaCl) salt because their host alkaliphilic bacteria also exhibit halophilic characteristics. It was noted that the protein from *D. hydrogenovorans* exhibited a conventional two-state unfolding curve only under high salt conditions (500 mM NaCl). Under the low salt condition, the two alkaliphilic NDKs from Bacillus sp. displayed the highest *T*_m_ at pH 7.0 and their *T*_m_s at pH 9.0 were higher than those at pH 5.0. Moreover, in the presence of 500 mM NaCl, the *T*_m_s of the three alkaliphilic NDKs at pH 9.0 were similar to those at pH 7.0 and their *T*_m_s at pH 5.0 were 14–23 °C lower than those at pH 9.0.

### Thermal stabilities of ancestral NDKs as a function of pH

We previously constructed phylogenetic trees comparing the amino acid sequences of NDKs in currently existing archaea and bacteria^12^. This enabled the reconstruction of various ancestral NDKs representing proteins in the common ancestor of archaea and that of bacteria. All the reconstructed NDKs appeared to be highly thermostable at near neutral pH. In this study, some of the resurrected NDKs (Arc3, Arc4, Arc5, Bac3, Bac4, Bac5) were subjected to temperature-induced unfolding experiments at pH values of 5.0, 7.0, and 9.0 (Fig. 1, Table 1). The six ancestral NDKs showed the highest *T*_m_ values at pH 7.0. Moreover, the *T*_m_ values of the ancestral NDKs at pH 9.0 were similar to those at pH 7.0. However, while Arc4 and Bac4 still exhibited high *T*_m_ values at pH 5.0, the *T*_m_ values of Arc3, Arc5, Bac3, and Bac5 at pH 5.0 were significantly lower than those observed at pH 7.0 and pH 9.0. Thus, the ancestral NDKs maintain greater thermostability in alkaline conditions, while their thermostabilities tend to decrease in acidic conditions. We conclude that the pH-dependencies of thermal stability for the reconstructed ancestral NDKs are more akin to those of NDKs from extant alkaliphilic bacteria rather than those from acidophilic or neutralophilic microorganisms.

The amino acid composition, ratio of acidic to basic amino acids and isoelectric points of the extant and ancestral NDKs are summarized in Tables S1 and S2. It has been reported that alkali-tolerant proteins often have increased proportions of arginine and histidine residues and a paucity of glutamate residues^44^. However, such a trend was not found in the extant alkaliphilic and ancestral NDKs. It is important to note that this observation specifically applies to the ancestral and alkaliphilic NDKs analyzed in this study. To ascertain the universality of this trend across all NDKs, additional analysis encompassing a more diverse set of NDKs is required. Alkaliphilic proteins are also known to have higher isoelectric points than their neutralophilic counterparts^45^. However, this was not the case for the alkaliphilic and ancestral NDKs because they have a greater ratio of acidic amino acids to basic amino acids. Consequently, the isoelectric point of alkaliphilic and ancestral NDKs is more acidic than the neutralophilic and acidophilic NDKs. Accordingly, the alkali-tolerance displayed by alkaliphilic NDKs and ancestral NDKs may involve a unique mechanism. Further mutational and structural analyses, including X-ray crystallography and molecular dynamics simulations, will be required to elucidate the precise mechanism of alkali tolerance of NDKs.

### Specific activities of ancestral and extant NDKs as a function of pH

**Figure 2:**
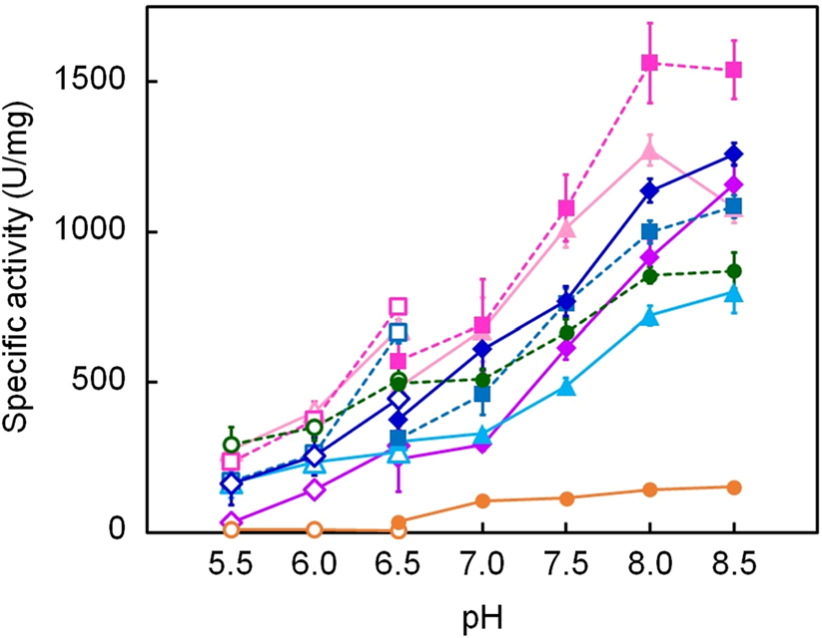
pH dependence of the specific activities of *S. tokodaii*, *A. fulgidus*, and ancestral NDKs. The specific activities were measured using a reaction mixture containing 5 mM each of ADP and GTP. Each value is the average of at least three independent measurements. Orange circles and solid lines, *S. tokodaii* NDK; green circles and dashed lines, *A. fulgidus* NDK; light pink triangles and solid lines, Arc3; pink squares and dashed lines, Arc4; purple diamonds and solid lines, Arc5; cyan triangles and solid lines, Bac3; blue squares and dashed lines, Bac4; dark blue diamonds and solid lines, Bac5.

The enzymatic activity of ancestral NDKs was measured by monitoring the transfer of phosphate from GTP to ADP to produce GDP and ATP, respectively. Figure 2 shows the pH dependence of the specific activities of the ancestral NDKs, as well as those of NDKs from extant acidophilic archaeon *S. tokodaii* and neutralophilic archaeon *A. fulgidus* for comparison. Specific activity increased with pH for NDKs from both *S. tokodaii* and *A. fulgidus*. However, the optimal pH for growth of *S. tokodaii* and *A. fulgidus* is pH 2–3 and approximately 7, respectively. Similarly, the ancestral NDKs functioned optimally in an alkaline solution. Thus, the pH profile of the specific activity of NDKs that originate from microorganisms inhabiting different pH environments did not correlate with the pH environments of the host microorganisms. In other words, the reaction catalyzed by NDK, transferring the γ-phosphate of GTP to ADP to generate GDP and ATP, showed its maximum efficiency at pH 8.0–8.5 independent of the optimal growth pH of the host microorganism. The observation of the highest reaction rate under alkaline conditions may be attributed more to the characteristics of the phosphotransfer reaction between a nucleoside triphosphate and a nucleoside diphosphate than to the nature of the catalyzing enzyme.

### Thermal stabilities of extant uS8s as a function of pH

**Figure 3:**
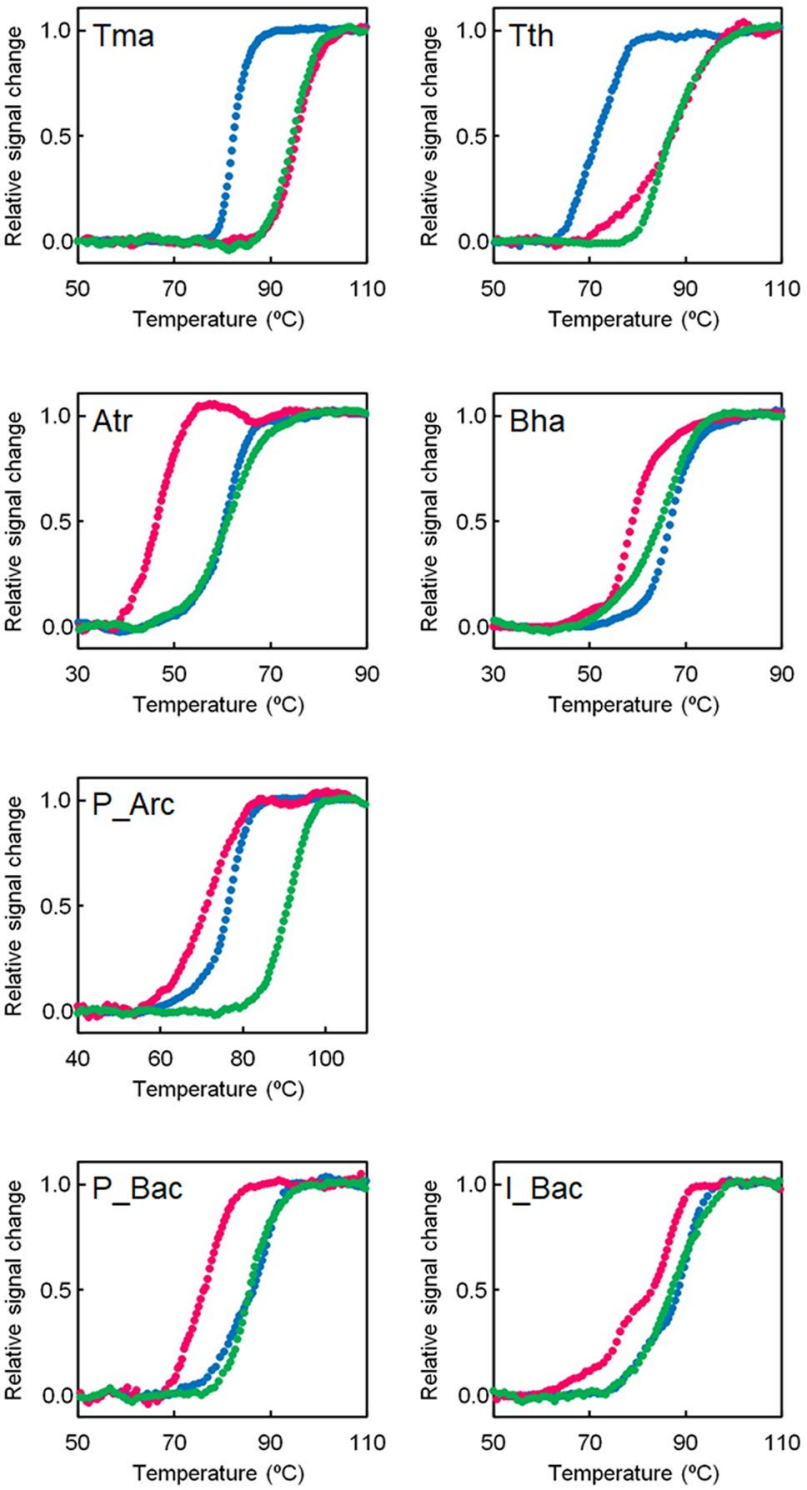
Temperature-induced unfolding curves of extant and ancestral uS8s at pH 5.0 (magenta), pH 7.0 (green) and pH 9.0 (blue). The change in ellipticity at 222 nm was monitored as a function of temperature. Within experimental error, duplicated measurements were identical. The plots were normalized with respect to the baseline of the native and denatured states. Tma, *T. maritima* uS8; Tth, *T. thermophilus* uS8; Atr, *A. transvaalensis* uS8; Bha, *B. halodurans* uS8.

The pH-dependent thermal stabilities of extant and ancestral uS8s were measured (Fig. 3; Table 2). For extant uS8s, we chose to analyze four proteins in all: namely, two from extant neutralophilic bacteria and two from alkaliphilic bacteria. For neutralophilic uS8s, we chose to analyze proteins from *T. thermophilus* and *Thermotoga maritima*. *T. maritima* is a hyperthermophilic bacterium that optimally grows at pH 7^46^. For both uS8s, the *T*_m_ values at pH 5.0 and pH 7.0 were similar or the same but the *T*_m_ values decreased at pH 9.0. Therefore, the extant uS8s from neutralophilic bacteria are less stable in alkali conditions compared to acidic and neutral conditions. For alkaliphilic uS8s, we chose to analyze proteins from *Alkaliphilus transvaalensis* and *B. halodurans*. *A. transvaalensis* grows in highly alkaline environments, with a pH range of 8.5 to 12.5 and optimally at pH 10.0^47^. In contrast to the neutralophilic uS8s, proteins from alkaliphilic bacteria are adapted to alkali conditions. Indeed, their *T*_m_ values at pH 9.0 were the same or even higher than those at pH 7.0. Moreover, their *T*_m_ values at pH 5.0 were significantly lower than those at pH 7.0 and pH 9.0.

**Table 2:** *T*_m_ (°C) for ancestral and extant uS8s.

Proteins	*T*_m_ ( °C)		
	pH 5.0	pH 7.0	pH 9.0
Ancestral uS8s			
P_Arc	71	92	79
P_Bac	76	87	87
I_Bac	83	87	88
Neutralophilic microbial uS8s			
*Tma* uS8	96	95	82
*Tth* uS8	87	87	72
Alkaliphilic microbial uS8s			
*Atr* uS8	47	61	61
*Bha* uS8	59	65	67

*T*_m_ values were estimated from the data shown in Fig. 3.

Tma *T. maritima*, Tth *T. thermophilus*, Atr *A. transvaalensis*, Bha *B. halodurans.*

### Thermal stabilities of ancestral uS8s as a function of pH

Previously, we built a phylogenetic tree containing the sequences of extant bacterial and archaeal uS8s, which was then used to infer the two last bacterial common ancestral sequences of uS8 (P_Bac and I_Bac)^36^. In this study, we inferred the last archaeal common ancestral sequences of uS8 using the same phylogenetic tree with either IQ-TREE^48^ or CodeML in PAML^49^. The archaeal sequence inferred using IQ-TREE was named I_Arc and that predicted by CodeML was named P_Arc (Supplementary Data 1). Genes encoding the two inferred sequences were synthesized and then used to prepare the ancestral uS8 of the last archaeal ancestor. However, I_Arc was not used in subsequent analyses as it precipitated during the purification process.

Temperature-induced unfolding experiments were performed at pHs 5.0, 7.0, and 9.0 for P_Arc, as well as for P_Bac and I_Bac. The unfolding curves are given in Fig. 3 and their *T*_m_s are listed in Table 2. P_Arc showed the highest *T*_m_ value at pH 7.0 and its *T*_m_ at pH 9.0 was higher than that at pH 5.0. Moreover, *T*_m_ values at pH 9.0 for P_Bac and I_Bac were similar to those at pH 7.0. However, the *T*_m_ values at pH 5.0 were significantly lower than those at pH 7.0 and pH 9.0. Therefore, akin to the results for ancestral NDKs, the structural stabilities of the ancestral uS8s are adapted to alkaline rather than acidic conditions. Consequently, the pH profiles of thermal stability for ancestral uS8s from the last bacterial common ancestor are similar to those of uS8s from extant alkaliphilic bacteria.

The amino acid composition, ratio of acidic to basic amino acids and isoelectric points of the extant and ancestral uS8s are summarized in Tables S3 and S4. Extant alkaliphilic uS8s have a smaller ratio of basic residues to acidic residues by comparison to neutralophilic uS8s. By contrast, ancestral uS8s have a larger ratio of basic residues. However, neutralophilic, alkaliphilic and ancestral uS8s all possess similar isoelectric points. As with NDK, further structural analysis will be required to reveal the molecular mechanism of alkali-tolerance in uS8s.

## Discussion

**Figure 4:**
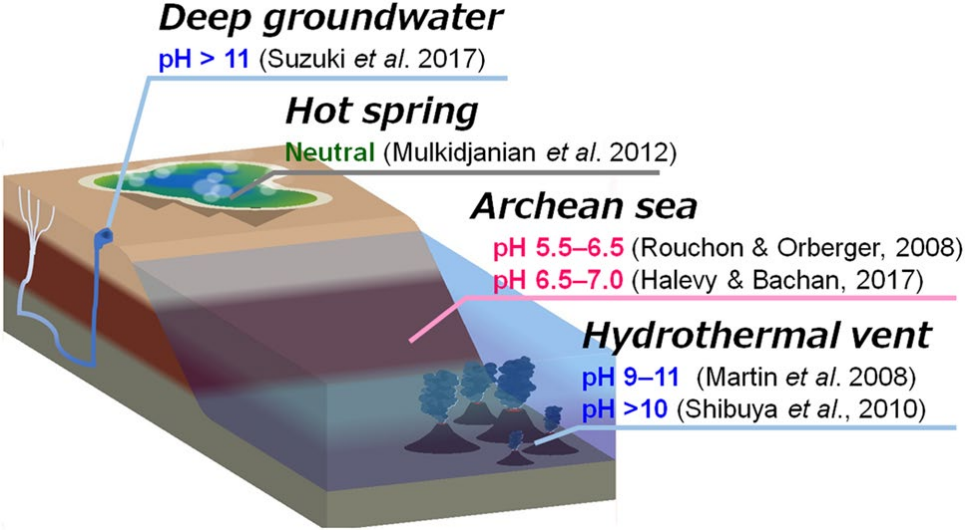
Candidate habitats for primitive life and their estimated pH values.

The potential of hydrogen or pH, which represents the hydrogen ion concentration in an aqueous solution, is a fundamental property of Earth's oceans and plays a crucial role in understanding water biogeochemistry. The primordial pH environment is especially significant as it played a vital role in the development of early life on Earth by providing the necessary conditions for the formation of organic compounds, including amino acids and nucleotides. Figure 4 illustrates candidate primordial biosphere environments and their estimated pH values. Several studies have attempted to estimate the pH of the early ocean. Rouchon and Orberger suggested that the early ocean was an acidic environment, with pH values ranging from 5.5 to 6.5 due to the dissolution of carbon dioxide from the atmosphere into the ocean^50^. Meanwhile, another study proposed that the early Earth's ocean may have had a more neutral pH environment, with pH values ranging from ~ 6.5 to 7.0^51^. This conclusion was reached using a statistical model of seawater pH as a function of atmospheric carbon dioxide pressure. Local environments that may have been the site of the origin and early evolution of life include submarine hydrothermal vents. Some of the submarine hydrothermal vents spew alkaline hydrothermal water, generating an environment with an extremely high pH value that could have potentially supported early life on primitive Earth^52,53^.

Aside from the ocean, terrestrial hot springs are another possible biosphere for primordial organisms. Some researchers have proposed that the origin of life relied on a land-based volcanic hot spring pool^54^. Koonin and colleagues predicted that primitive hot springs were neutral^55^. Deep groundwater could also provide a possible habitat for primitive life. Water that springs from the ultramafic rocks and serpentinite soils at The Cedars was once thought to be too harsh for life due to its highly alkaline nature (pH =  ~ 12) and highly reducing conditions. However, Suzuki et al. discovered that this seemingly harsh and challenging environment supports a surprisingly diverse range of microbes with specialized metabolic strategies^56^. Such springs, which rely on deep-seated water as a source, might have provided a habitat for primitive biotic communities.

The technique of ASR allows us to reconstruct proteins from ancient organisms^2,6,57,58^. In the present study, this method was employed to obtain insights into the likely pH of primitive habitats. Each protein has an optimal pH range in which it is most stable and functional. This is because the amino acid side chains within the protein have different p*K*_a_ values, which determine their ionization state at different pHs. When the pH of the environment does not correspond to the optimal range for the protein, it can alter the charged state of these amino acid residues and destabilize the protein fold. The pH range that promotes protein stability is thought to reflect the intracellular pH of the organism hosting the protein. It is known that certain acidophilic microorganisms possess acidic cytosols, necessitating the functionality of the proteome at low pH levels^59^. Alternatively, other acidophilic microorganisms maintain a cytoplasmic pH close to neutrality by balancing protons and electrons through the use of transporters and channels, thereby obviating the necessity for the proteome to function at low pH^60^. Conversely, organisms living in alkaline environments have higher intracellular pH, although the intracellular pH values are usually more neutral than the external environment^61^. Therefore, microorganisms inhabiting alkaline environments need to possess proteins that are sufficiently stable and functional at high pH.

Most of the reconstructed ancestral proteins examined in this study were found to be more stable in alkaline conditions, displaying properties similar to those from contemporary alkaliphilic bacteria. Specifically, the ancestral NDKs, Arc4 and Bac4, and the bacterial common ancestral uS8s, P_Bac, and I_Bac, exhibited comparable thermal stability at both alkaline and neutral pHs (Figs. 1 and 3; Tables 1 and 2). By contrast, microbial proteins derived from acidophilic and neutralophilic organisms displayed higher unfolding temperatures at pH 5.0 than at pH 9.0 (Figs. 1 and 3; Tables 1 and 2). The findings suggest that the bacterial and archaeal common ancestors may have thrived in an alkaline environment such as submarine alkaline hydrothermal vents or alkaline springs originating from deep groundwater.

In the presence of NaCl, protein stability is known to vary—either improving, decreasing, or remaining unchanged—depending on the specific protein^62–64^. However, our understanding of salinity in the primordial environment is limited. Blaber and colleagues demonstrated the necessity of a halophilic environment for the folding of proteins designed with a bias for prebiotic amino acids, presumed to have been available in the early Earth's environment^65^. Their study emphasizes the significance of a halophilic environment in the context of early protein synthesis and, consequently, in abiotic events.

Our temperature-induced unfolding experiment using alkaliphilic NDKs demonstrated a stabilizing effect of 500 mM NaCl at pH 9.0 (Fig. 1; Table 1). Similarly, it is plausible that salinity levels played a crucial role in maintaining the stability of the cellular machinery of ancestral microorganisms. However, an unfolding experiment conducted on the ancestral NDK, Arc3, in the presence of 500 mM NaCl at pH 9.0 yielded an atypical thermal unfolding curve (Fig. S2), and thus the unfolding temperature could not be estimated. Therefore, thermal unfolding experiments under the presence of 500 mM NaCl for other ancestral NDKs and acidophilic/neutralophilic NDKs were not performed. Additionally, for uS8, the unfolding temperature was consistently measured under the presence of 250 mM NaCl, as analysis at lower salt concentrations was not feasible due to precipitation. The salinity levels in the primordial biosphere environment need further investigation using other proteins in future studies.

ASR relies on a phylogenetic tree of homologous amino acid sequences. However, obtaining an accurate tree and faithfully reconstructing ancestral sequences is technically challenging. Although advancements in ASR techniques over the past decade have greatly improved the accuracy, implications drawn from the tree and ancestral sequences are difficult to verify. Several studies have used ASR to estimate the temperature of the ancient biosphere, which suggested the thermophilic ancestry of life based on the high thermostability of most reconstructed ancestral proteins^12,23^. However, Williams et al. proposed that inaccurately reconstructed sequences could result in an overestimation of thermostability^66^. Tawfik and colleagues also suggested that high thermodynamic stability of ancestral proteins may have been driven by other factors, such as high oxidative pressure and radiation levels, absence of cellular osmolytes or chaperones, or the low fidelity of transcription–translation machinery^67^. ASR has also been used to connect ancient proteins to early environments. One such study was conducted by Kaçar and colleagues, who used ASR to resurrect an ancestral RuBisCO gene from extant cyanobacteria^68^. When cultured in conditions corresponding to the potential Precambrian environment the carbon isotope signatures of the engineered cyanobacteria were within modern ranges. Although this finding has important implications for understanding the uniformitarian assumptions of carbon isotope signatures over geologic time, they also noted that the modern organism and its proteins may have influenced the ancestral RuBisCO phenotype. The same group also warned of the potential pitfalls of interpreting paleophenotype models and data^25^. Therefore, despite the proven usefulness of ASR for linking ancient proteins to early environments, it is crucial to consider the limitations of ASR, such as the potential for errors in building phylogenetic trees and inferring ancestral sequences.

Prior to this study, it has been widely accepted that ASR provides a powerful approach for generating thermally stable proteins. Our present results expand upon this knowledge by demonstrating that ASR is also an effective method for producing alkali-tolerant proteins. Such proteins have significant potential for use in diverse fields, such as industrial applications and bioremediation, due to their ability to remain stable at high pHs and function even in the presence of detergents^44^. Furthermore, they are valuable in instances where the pH-enhanced solubility of substrates and ligands is anticipated^44^. In the realm of environmental science, alkali-tolerant enzymes may, in principle, be useful tools for the remediation of alkaline soils and wastewater. Given that ASR can produce proteins that are stable against both heat and alkali, this method is poised to become a valuable technique for generating proteins that can be used in a variety of contexts.

## Methods

### Preparation of ancestral and extant NDKs

The expression plasmids used for ancestral NDKs (Arc3, Arc4, Arc5, Bac3, Bac4, Bac5) and extant NDKs from *T. thermophilus* and *A. fulgidus* were constructed previously^12^. The genes encoding extant NDKs from *M. jannaschii* and *S. tokodaii* were synthesized by Eurofins Genomics (Tokyo, Japan) and then cloned into the *Nde*I-*Bam*HI restriction recognition sites of plasmid pET23a(+) (Merck, Tokyo, Japan). The genes encoding extant NDKs from alkaliphilic microorganisms*, B. halodurans*, *B. pseudofirmus* and *D. hydrogenovorans* were also synthesized by Eurofins Genomics and then cloned into the *Nde*I-*Bam*HI restriction recognition sites of plasmid pET15b (Merck) so that the resulting recombinant proteins include an N-terminal polyhistidine tag. To prepare and purify each of the ancestral and extant NDKs, one of the expression plasmids were used to transform *Escherichia coli* Rosetta 2(DE3) (Merck) and the resulting transformant was cultured in Luria–Bertani (LB) medium supplemented with 150 μg/ml ampicillin. Heterologous gene expression was induced using the Overnight Express Autoinduction system I (Merck). After overnight culture at 37 °C, cells were harvested and disrupted by sonication. The soluble fraction was recovered after centrifugation at 60,000 × *g* for 20 min. To purify the ancestral and extant thermophilic (*T. thermophilus, A. fulgidus*, *M. jannaschii*, *S. tokodaii*) NDKs, the supernatants were heated at 80 °C for 20 min and centrifuged at 60,000 × *g* for 20 min to precipitate the denatured *E. coli* proteins. The supernatants were then subjected to chromatography using a HiTrap Q and Resource Q column (Cytiva, Tokyo, Japan). Alkaliphilic NDKs were purified by chromatography using a HisTrap HP and Resource Q column (Cytiva). All the resulting protein preparations appeared to be homogeneous as judged by sodium dodecyl sulfate–polyacrylamide gel electrophoresis followed by Coomassie blue staining.

### Thermal stability measurements of NDKs at various pHs

The A_280_ values of the protein solutions were used to determine their concentrations. The molar absorption coefficient at 280 nm for each protein was determined using a modified method of Pace and colleagues^69^ based on the procedure described by Gill and von Hippel^70^. The thermal denaturation profile for each protein was monitored by observing the change in ellipticity at 222 nm using a J-1100 spectropolarimeter (Jasco, Tokyo, Japan) equipped with a programmable temperature controller and a pressure-proof cell compartment to prevent bubbling and evaporation at high temperatures. Protein solutions were diluted to a final concentration of 20 μM with 20 mM potassium citrate (pH 5.0), potassium phosphate (pH 7.0) or potassium borate (pH 9.0), supplemented with 50 mM KCl and 1 mM EDTA. Thermal denaturation of the extant alkaliphilic NDKs was also measured in the solution supplemented with 500 mM NaCl. A cell with a pathlength of 0.1 cm was used, and the temperature was increased at a rate of 1.0 °C/min.

### Specific activity measurements of NDKs at various pHs

Enzymatic activity was determined at 70 °C by measuring the increase in the amount of product ATP using the Kinase-Glo Luminescent Kinase Assay kit (Promega, Tokyo, Japan). The assay solution contained 50 mM MOPS (pH 5.5–6.5) or HEPES (pH 6.5–8.5), 25 mM KCl, 10 mM (NH_4_)_2_SO_4_, 2.0 mM Mg(CH_3_COO)_2_, 1.0 mM dithiothreitol, 5.0 mM ADP, and 5.0 mM GTP. One unit of enzyme activity was defined as the formation of 1 μmol of ATP per minute.

### Ancestral sequence inference of the last archaeal common ancestor of uS8

We previously inferred two amino acid sequences of the last bacterial common ancestral uS8 based on a phylogenetic tree consisting of extant archaeal and bacterial uS8 sequences^36^. In this study, two amino acid sequences of the last archaeal common ancestor were inferred using the same phylogenetic tree and either IQ-TREE^48^ or CodeML in PAML^49^. GASP^71^ was used to estimate the location of gaps in the ancestral sequences. The resulting amino acid sequences were named I_Arc and P_Arc, which are available in FASTA format (Supplementary Data 1).

### Construction of expression plasmids for ancestral and extant uS8

The genes encoding archaeal ancestral uS8s were synthesized by Eurofins Genomics. The genes encoding extant uS8 from *T. thermophilus*, *T. maritima*, *A. transvaalensis* and *B. halodurans* were also synthesized by Eurofins Genomics. To construct I_Arc and P_Arc carrying a GST-tag at its N-terminus, the respective genes were ligated into pGEX-6p-1 (Cytiva). The genes encoding extant alkaliphilic uS8s from *A. transvaalensis* and *B. halodurans* were also cloned into pGEX-6p-1. The gene encoding uS8s from extant neutralophilic thermophiles, *T. thermophilus* and *T. maritima*, were cloned into pET23a(+) (Merck).

### Purification of uS8s

The bacterial ancestral uS8s were overexpressed in *E. coli* and subsequently purified as described previously^36^. The archaeal ancestral and the extant alkaliphilic uS8s were expressed and purified as an N-terminally GST-tagged form. For their production, *E. coli* Rosetta 2(DE3) was transformed with the respective expression plasmids and then cultured in LB medium supplemented with 150 μg/ml ampicillin. Overexpression was induced with Overnight Express Autoinduction System 1. After overnight cultivation, cells were harvested by centrifugation and disrupted by sonication. The precipitate was removed by centrifugation at 60,000 × g for 20 min and the soluble proteins were subjected to GSTrap HP affinity column chromatography (Cytiva). The N-terminal GST-tag (excepting residues G_-4_P_-3_L_-2_G_-1_) was then removed by digestion with PreScission Protease (Cytiva). The PreScission Protease and newly liberated GST-tag were subsequently removed by gel filtration chromatography on a Superdex 75 column (Cytiva). For preparation of *T. thermophilus* and *T. maritima* uS8s, *E. coli* Rosetta 2(DE3) carrying the expression plasmid was cultivated in LB medium supplemented with 150 μg/ml ampicillin. Expression was induced with Overnight Express Autoinduction System 1 (Merck). After overnight culture, cells were harvested by centrifugation and disrupted by sonication. The supernatants were then subjected to cation exchange chromatography on a HiTrap SP-FF column (Cytiva). All the purified proteins appeared to be homogeneous as judged by sodium dodecyl sulfate–polyacrylamide gel electrophoresis followed by Coomassie blue staining.

### Thermal stability measurements of uS8s at various pH values

The A_280_ value of each purified protein was measured, and the concentration was then calculated using the respective extinction coefficient. To record the thermal denaturation profile of each protein, the change in ellipticity at 222 nm was monitored using a J-1100 spectropolarimeter (Jasco), which was equipped with a programmable temperature controller and a pressure-proof cell compartment. The protein solutions were diluted with 20 mM potassium citrate (pH 5.0), potassium phosphate (pH 7.0), or potassium borate (pH 9.0), supplemented with 250 mM NaCl and 1 mM EDTA, to achieve a final concentration of 20 μM. A 0.1 cm pathlength cell was used, and the temperature was increased at a rate of 1.0 °C/min.

### Supplementary Information

Supplementary Information.

## Data Availability

All data is included in the manuscript and/or supplementary materials.
