# Supplementary material for: Comparative analysis of reconstructed ancestral proteins with their extant counterparts suggests primitive life had an alkaline habitat
Source: Sci Rep. 2024 Jan 3;14:398. doi: 10.1038/s41598-023-50828-4 (PMC10764835; doi:10.1038/s41598-023-50828-4)
Supplement: Supplementary file 1 — Supplementary Information. [file 41598_2023_50828_MOESM1_ESM.pdf]

Supplementary materials for

Comparative analysis of reconstructed ancestral proteins with their extant counterparts suggests primitive life had an alkaline habitat

Takayuki Fujikawa, Takahiro Sasamoto, Fangzheng Zhao, Akihiko Yamagishi, Satoshi Akanuma

Corresponding author: Satoshi Akanuma
Email: akanuma@waseda.jp

This PDF file includes:

Figures S1 and S2
Tables S1 to S4
Supplementary Data 1

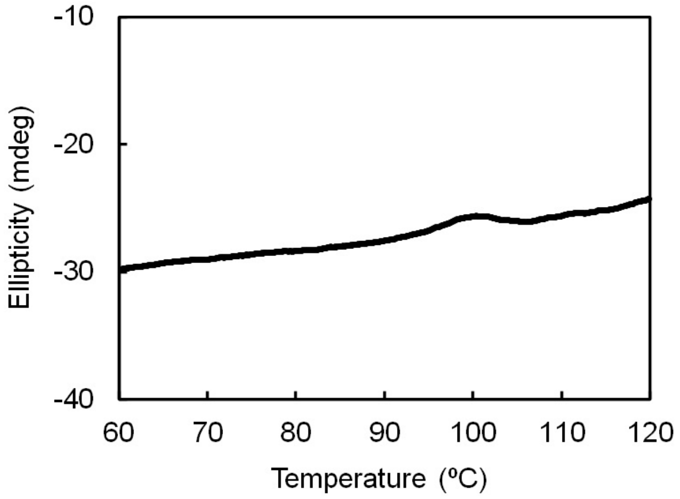

Figure S1. A representative unfolding curve observed for *A. fulgidus* NDK at pH 7.0. The raw data for the change in ellipticity at 222 nm is presented as a function of temperature. The scan rate was 1.0ºC/min. The sample comprised 20 μM protein in 20 mM potassium phosphate (pH 7.0), 50 mM KCl, 0.5 mM EDTA.

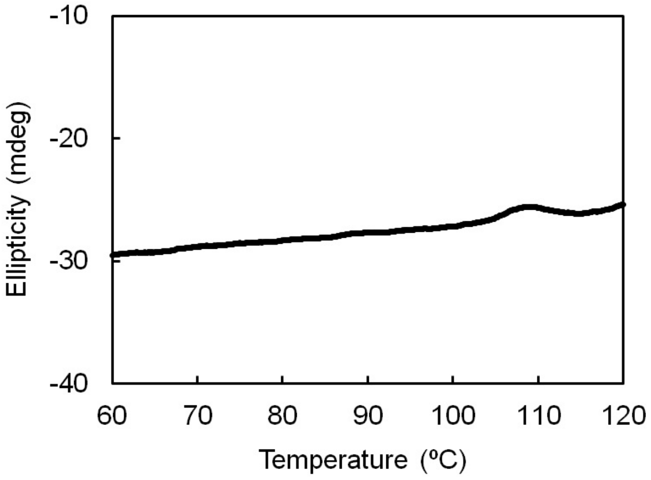

Figure S2. An unfolding curve observed for Arc3 at pH 9.0 in the presence of 500 mM NaCl. The raw data for the change in ellipticity at 222 nm is presented as a function of temperature. The scan rate was 1.0ºC/min. The sample comprised 20 μM protein in 20 mM potassium borate (pH 9.0), 500 mM NaCl, 0.5 mM EDTA.

Table S1. Isoelectric point (pI), ratio of acidic (Asp, Glu) and basic (Lys, Arg) residues, and amino acid composition of ancestral and extant NDKs.

	Arc3	Bac3	Arc4	Bac4	Arc5	Bac5	Sto[a]	Tth[a]	Mja[a]	Bha[a]	Bps[a]	Dhy[a]
pI	5.65	5.25	5.41	5.65	5.09	5.24	6.85	6.92	6.77	5.11	5.24	5.01
acidic/basic	1.16	1.28	1.22	1.16	1.35	1.29	1.00	1.00	1.00	1.35	1.31	1.36
Asp	9	9	9	9	9	8	6	5	7	8	6	8
Glu	13	14	13	13	14	14	17	14	19	15	15	11
Lys	10	9	9	9	7	8	13	6	17	9	9	9
Arg	9	9	9	10	10	9	10	13	9	8	7	5
His	3	3	3	3	3	3	3	4	2	3	3	2
Ser	8	8	7	7	10	10	10	3	5	7	6	9
Thr	4	4	4	4	4	4	6	7	6	9	10	6
Asn	3	3	5	4	3	3	3	1	5	6	7	5
Gln	2	2	2	2	2	2	2	3	0	3	2	3
Tyr	3	2	3	2	2	2	3	1	3	3	4	4
Gly	11	11	10	11	10	11	10	12	8	12	13	13
Pro	6	6	6	6	6	7	7	8	4	6	6	6
Cys	0	0	0	0	0	0	1	0	0	0	0	2
Ala	12	12	13	12	12	11	8	13	10	11	12	14
Val	14	13	15	13	15	13	12	12	8	13	15	9
Leu	6	7	7	9	6	7	8	11	6	6	7	9
Ile	12	12	11	10	11	11	15	9	14	10	8	12
Met	7	7	6	7	7	8	8	6	8	7	7	5
Phe	7	8	7	8	8	8	6	9	9	9	8	7
Trp	0	0	0	0	0	0	0	0	0	2	2	0
Total	139	139	139	139	139	139	148	137	140	147	147	139

[a] Sto, *S. tokodaii* NDK; Mja, *M. jannaschii* NDK; Tth, *T. thermophilus* NDK; Bha, *B. halodurans* NDK; Bps, *B. pseudofirmus* NDK; Dhy, *D. hydrogenovorans* NDK.

Table S2. Percentage amino acid composition of ancestral and extant NDKs.

	Arc3	Bac3	Arc4	Bac4	Arc5	Bac5	Sto[a]	Tth[a]	Mja[a]	Bha[a]	Bps[a]	Dhy[a]
Asp	6.47	6.47	6.47	6.47	6.47	5.76	4.05	3.65	5.00	5.44	4.08	5.76
Glu	9.35	10.1	9.35	9.35	10.1	10.1	11.5	10.2	13.6	10.2	10.2	7.91
Lys	7.19	6.47	6.47	6.47	5.04	5.76	8.78	4.38	12.1	6.12	6.12	6.47
Arg	6.47	6.47	6.47	7.19	7.19	6.47	6.76	9.49	6.43	5.44	4.76	3.60
His	2.16	2.16	2.16	2.16	2.16	2.16	2.03	2.92	1.43	2.04	2.04	1.44
Ser	5.76	5.76	5.04	5.04	7.19	7.19	6.76	2.19	3.57	4.76	4.08	6.47
Thr	2.88	2.88	2.88	2.88	2.88	2.88	4.05	5.11	4.29	6.12	6.80	4.32
Asn	2.16	2.16	3.60	2.88	2.16	2.16	2.03	0.73	3.57	4.08	4.76	3.60
Gln	1.44	1.44	1.44	1.44	1.44	1.44	1.35	2.19	0.00	2.04	1.36	2.16
Tyr	2.16	1.44	2.16	1.44	1.44	1.44	2.03	0.73	2.14	2.04	2.72	2.88
Gly	7.91	7.91	7.19	7.91	7.19	7.91	6.76	8.76	5.71	8.16	8.84	9.35
Pro	4.32	4.32	4.32	4.32	4.32	5.04	4.73	5.84	2.86	4.08	4.08	4.32
Cys	0.00	0.00	0.00	0.00	0.00	0.00	0.68	0.00	0.00	0.00	0.00	1.44
Ala	8.63	8.63	9.35	8.63	8.63	7.91	5.41	9.49	7.14	7.48	8.16	10.1
Val	10.1	9.35	10.8	9.35	10.8	9.35	8.11	8.76	5.71	8.84	10.2	6.47
Leu	4.32	5.04	5.04	6.47	4.32	5.04	5.41	8.03	4.29	4.08	4.76	6.47
Ile	8.63	8.63	7.91	7.19	7.91	7.91	10.1	6.57	10.0	6.80	5.44	8.63
Met	5.04	5.04	4.32	5.04	5.04	5.76	5.41	4.38	5.71	4.76	4.76	3.60
Phe	5.04	5.76	5.04	5.76	5.76	5.76	4.05	6.57	6.43	6.12	5.44	5.04
Trp	0.00	0.00	0.00	0.00	0.00	0.00	0.00	0.00	0.00	1.36	1.36	0.00

[a] Sto, *S. tokodaii* NDK; Mja, *M. jannaschii* NDK; Tth, *T. thermophilus* NDK; Bha, *B. halodurans* NDK; Bps, *B. pseudofirmus* NDK; Dhy, *D. hydrogenovorans* NDK.

Table S3. Isoelectric point (pI), ratio of acidic (Asp, Glu) to basic (Lys, Arg) residues, and amino acid composition of ancestral and extant uS8s.

	I_Bac	P_Bac	P_Arc	Tth[a]	Tma[a]	Atr[a]	Bha[a]
pI	9.97	10.00	9.83	10.26	9.84	9.80	9.73
acidic/basic	0.467	0.464	0.533	0.586	0.577	0.652	0.696
Asp	7	6	5	7	8	7	5
Glu	7	7	11	10	7	8	11
Lys	23	20	24	9	15	15	12
Arg	7	8	6	20	11	8	11
His	0	0	1	2	3	1	1
Ser	5	7	5	5	4	4	8
Thr	4	3	5	6	5	7	6
Asn	7	8	5	1	5	6	3
Gln	2	3	0	2	2	2	4
Tyr	6	6	6	6	7	3	5
Gly	10	11	8	13	13	14	12
Pro	4	4	4	9	3	4	4
Cys	1	1	1	1	1	0	0
Ala	6	6	11	6	8	9	8
Val	11	10	6	13	11	13	11
Leu	10	8	7	11	9	10	11
Ile	14	15	16	12	15	15	13
Met	4	5	5	2	3	3	4
Phe	1	1	3	2	2	2	2
Trp	1	1	0	1	2	1	1
Total	130	130	129	138	134	132	132

[a] Tth, *T. thermophilus* uS8; Tma, *T. maritima* uS8; Atr, *A. transvaalensis* uS8; Bha, *B. halodurans* uS8.

Table S4. Percentage amino acid composition of ancestral and extant uS8s.

	I_Bac	P_Bac	P_Arc	Tth[a]	Tma[a]	Atr[a]	Bha[a]
Asp	5.38	4.62	3.88	5.07	5.97	5.30	3.79
Glu	5.38	5.38	8.53	7.25	5.22	6.06	8.33
Lys	17.7	15.4	18.6	6.52	11.2	11.4	9.09
Arg	5.38	6.15	4.65	14.5	8.21	6.06	8.33
His	0.00	0.00	0.78	1.45	2.24	0.76	0.76
Ser	3.85	5.38	3.88	3.62	2.99	3.03	6.06
Thr	3.08	2.31	3.88	4.35	3.73	5.30	4.55
Asn	5.38	6.15	3.88	0.72	3.73	4.55	2.27
Gln	1.54	2.31	0.00	1.45	1.49	1.52	3.03
Tyr	4.62	4.62	4.65	4.35	5.22	2.27	3.79
Gly	7.69	8.46	6.20	9.42	9.70	10.6	9.09
Pro	3.08	3.08	3.10	6.52	2.24	3.03	3.03
Cys	0.77	0.77	0.78	0.72	0.75	0.00	0.00
Ala	4.62	4.62	8.53	4.35	5.97	6.82	6.06
Val	8.46	7.69	4.65	9.42	8.21	9.85	8.33
Leu	7.69	6.15	5.43	7.97	6.72	7.58	8.33
Ile	10.8	11.5	12.4	8.70	11.2	11.4	9.85
Met	3.08	3.85	3.88	1.45	2.24	2.27	3.03
Phe	0.77	0.77	2.33	1.45	1.49	1.52	1.52
Trp	0.77	0.77	0.00	0.72	1.49	0.76	0.76

[a] Tth, *T. thermophilus* uS8; Tma, *T. maritima* uS8; Atr, *A. transvaalensis* uS8; Bha, *B. halodurans* uS8.

Supplementary Data 1. Amino acid sequences of ancestral NDKs and uS8s in FASTA format.

Ancestral NDKs

>Arc3
MERTFVMIKPDGVQRGLVGEIISRFERKGLKIVAMKMMQISRELAEKHYAEHKGKPFFDDLVDYIT
SGPVVAMVVEGKNAISVVRKMVGATNPAEAAPGTIRGDFALDIGRNVIHASDSPESAEREISLFFK
EDEIVDY

>Arc4
MERTFVMIKPDGVQRGLVGEIISRFERKGLKIVALKMMQISRELAEKHYAEHKDKPFFDDLVDYIT
SGPVVAMVVEGENAIAVVRKMVGATNPAKAAPGTIRGDFALDIGRNVVHASDSPESAEREISLFF
NEDEIVNY

>Arc5
MERTFVMIKPDGVQRGLVGEIISRFERKGLKIVAMKMMQISRELAEKHYAEHREKPFFDDLVDFIT
SGPVVAMVVEGENAISVVRKMVGATNPAEAAPGTIRGDFALSIGRNVVHASDSPESAEREISLFF
SDDEIVDY

>Bac3
MERTFVMIKPDGVQRGLVGEIISRFERKGLKIVAMKMMQISRELAEKHYAEHKGKPFFDDLVDFIT
SGPVVAMVLEGENAISVVRKMVGATNPAEAAPGTIRGDFALDIGRNVIHASDSPESAEREISLFFK
EDEIVDY

>Bac4
MERTFVMIKPDGVQRGLVGEIISRFERKGLKIVALKMMQISRELAEKHYAEHKDKPFFDDLVDFIT
SGPVVAMVLEGENAIAVVRKMMGATNPAKAAPGTIRGDFALDIGRNVVHGSDSPESAEREISLFF
REDELVNY

>Bac5
MERTFVMIKPDGVQRGLVGEIISRFERKGLKIVAMKMMQISRELAEKHYAEHKEKPFFDDLVDFIT
SGPVVAMVLEGENAISVVRKMMGATNPAEAAPGTIRGDFALSIGRNVVHGSDSPESAEREISLFF
SPDEIVDY

Ancestral uS8s

>I_Arc
MSRDPVADALTNIKNAEKAGKKEVTIKPASKLILEILKIMKKKGYIKKYKLIDDGREGMFKVELKGKI
NECKAIKPRFAVKKSEIEKYEKRYLPSKDIGILIVSTSKGVMTHKEAKEKNVGGRLIAYVY

>P_Arc
MSRDPVADALTNIKNAKMAAKEEVTIKPASKLITEILKIMKKKGYIKNYKFIDDGREGMFKIELKGKI
NECKAIKPRFAVKKSEIEKYEKRYLPARDIGILIVSTSKGVMTHKEAKEKNIGGRLIAYVY

>I_Bac
MSTDPIADMLTRIRNANKAMKEKVDIPASKLKLEILKILKKEGFIKDYKYIEDNKQGILRVYLKYGNK
KRVINGLKRVSKPGLRVYVKKDEIPKVKNGLGIAIISTSKGVMTDKEARQKNVGGEVICYVW

>P_Bac
MSSDPIADMLTRIRNANMAMKEKVDIPASKLKQEILKILKKEGFIKNYKYIEDNKQGILRVYLKYGN
NKRVINGLKRVSKPGRRVYVGKDEIPKVKSGLGIAIISTSKGIMTDKEARQKNVGGEVICYVW
